# Supplementary material for: Dynamic mechanisms of CRISPR interference by Escherichia coli CRISPR-Cas3
Source: Nat Commun. 2022 Aug 30;13:4917. doi: 10.1038/s41467-022-32618-0 (PMC9427990; doi:10.1038/s41467-022-32618-0)
Supplement: Supplementary file 1 — Supplementary Information [file 41467_2022_32618_MOESM1_ESM.pdf]

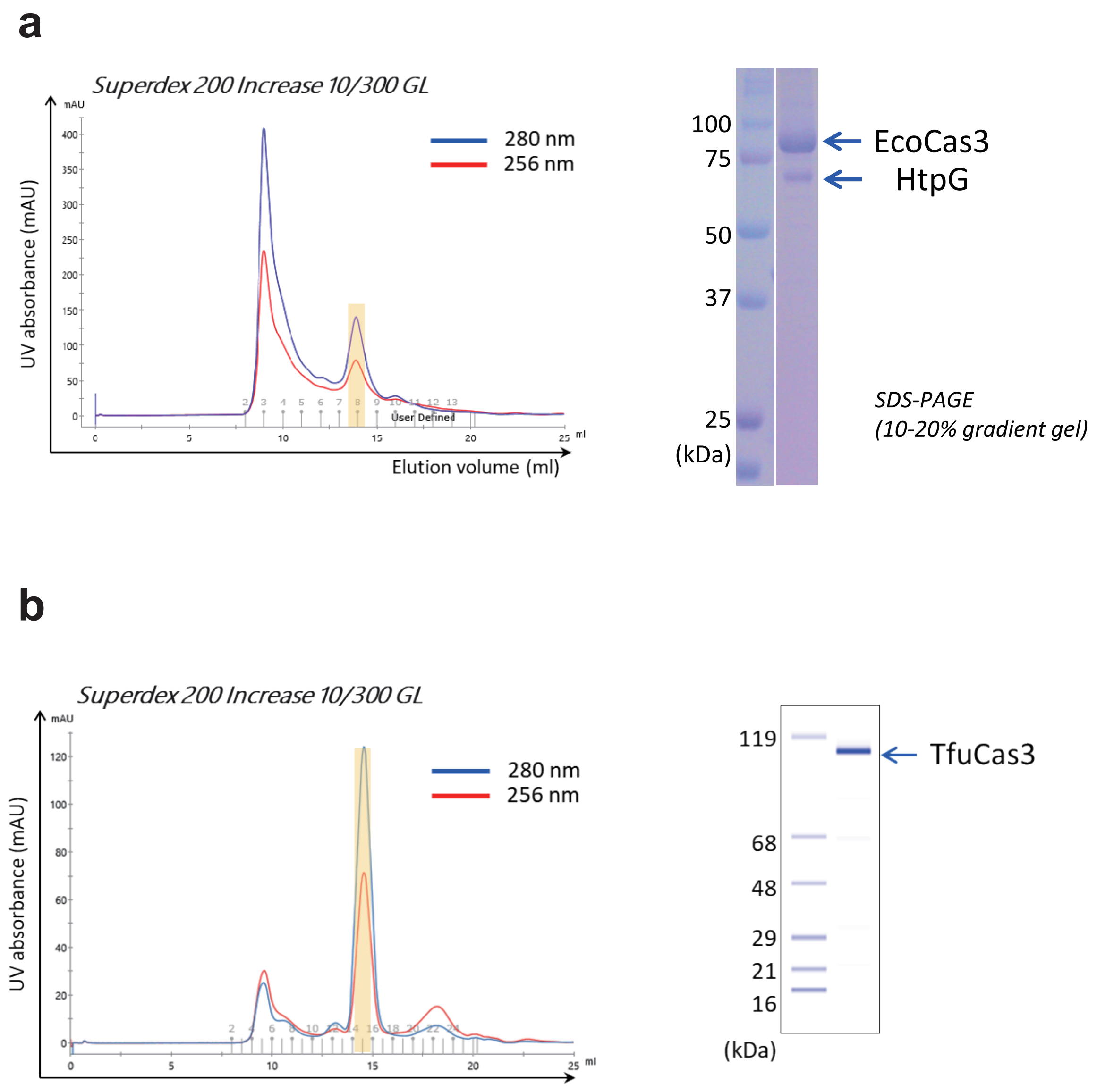

Supplementary Fig. 1. Purification of EcoCas3 and TfuCas3 recombinant proteins by the *E. coli* bacterial expression system. (a) A limited amount of highly aggregated *E. coli* Cas3 (EcoCas3) protein was purified by size-exclusion chromatography and was evaluated by sodium dodecyl sulfate-polyacrylamide gel electrophoresis (SDS-PAGE). Co-expression of HtpG chaperon and low temperature 20°C culture were used in the *E. coli* expression system. (b) Isolation of large amounts of *Thermobifida fusca* Cas3 (TfuCas3) protein at 37°C. This purification was repeated twice independently with similar results.

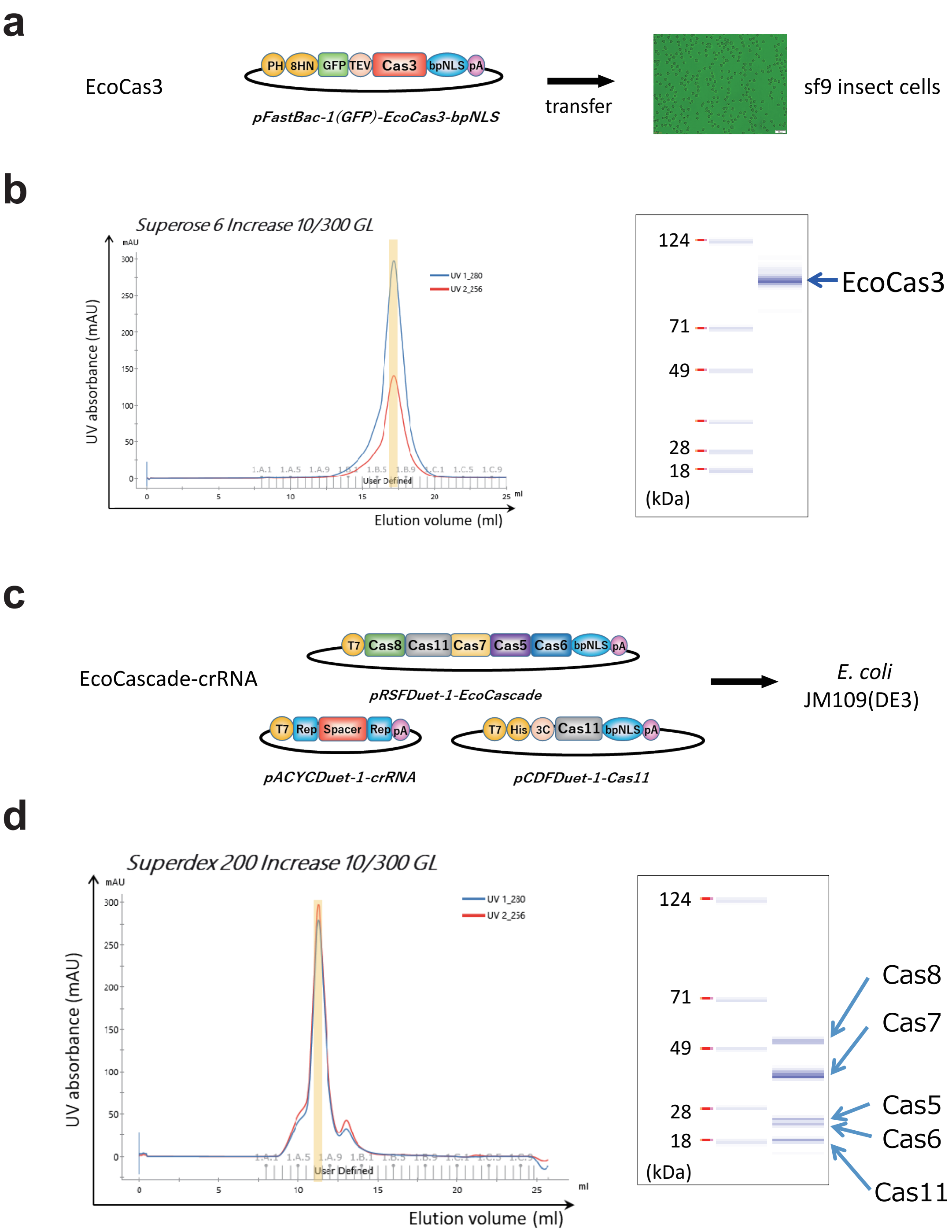

Supplementary Fig. 2. Purification of EcoCas3 and EcoCascade (Cas5, Cas6, Cas7, Cas8, and Cas11) ribonucleoproteins. (a) A large amount of EcoCas3 protein was purified using a baculovirus expression system in Sf9 insect cells cultured at 28°C. (b) Purified EcoCas3 protein from Sf9 cells was soluble and ~95% homogeneous on SDS-PAGE. (c,d) A complex of Cas5, Cas6, Cas7, Cas8, and Cas11 proteins and crRNA was co-expressed in JM109(DE3) ***E. coli*** cultured at 37°C, purified using Ni-NTA resin, and separated by size exclusion chromatography (SEC). These experiments were repeated three times independently with almost same results.

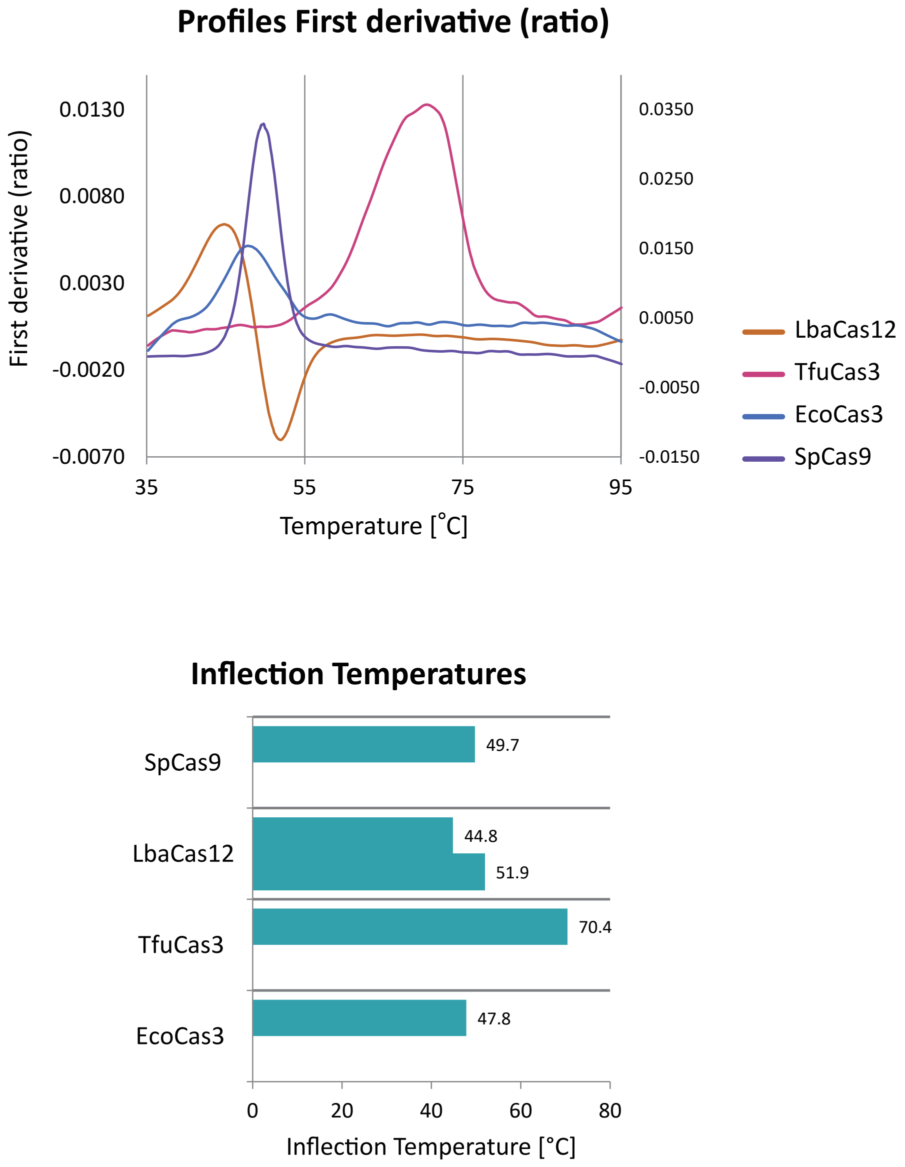

Supplementary Fig. 3. Temperature-dependent stability of recombinant EcoCas3 protein. To evaluate the temperature-dependent stability of purified EcoCas3, we employed a modified nanoscale differential scanning fluorimetry method, nanoDSF, which determines protein stability by measuring intrinsic tryptophan or tyrosine fluorescence using the Tycho NT.6 system (NanoTemper Technologies GmbH). The profiles show the first derivative ratio of 330 and 350 nm as the temperature rises from 35°C to 95°C. EcoCas3 protein purified from Sf9 cells had a temperature inflection point (Ti) of 47.8°C, while TfuCas3 and off-the-shelf SpCas9 proteins had Ti’ s of 70.4°C and 49.7°C, respectively. LbaCas12a protein had two Tis (44.8 and 51.9°C), which may represent dimeric structural dissociation between the REC and NUC lobes.

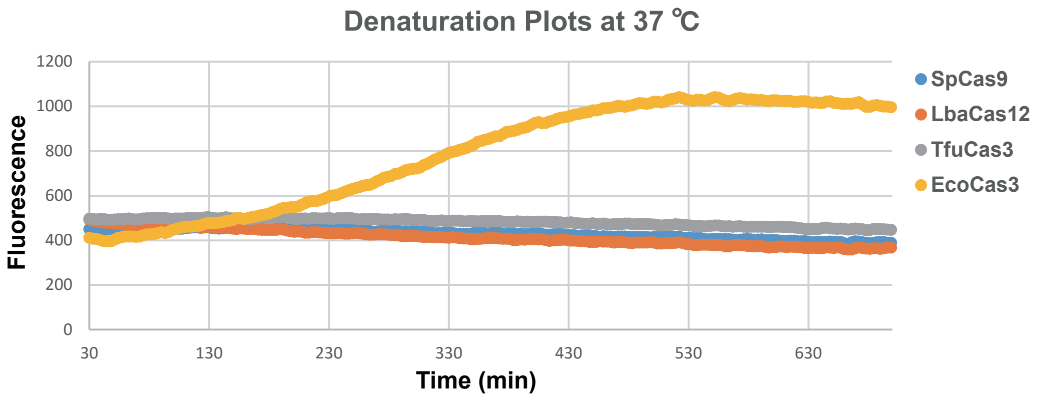

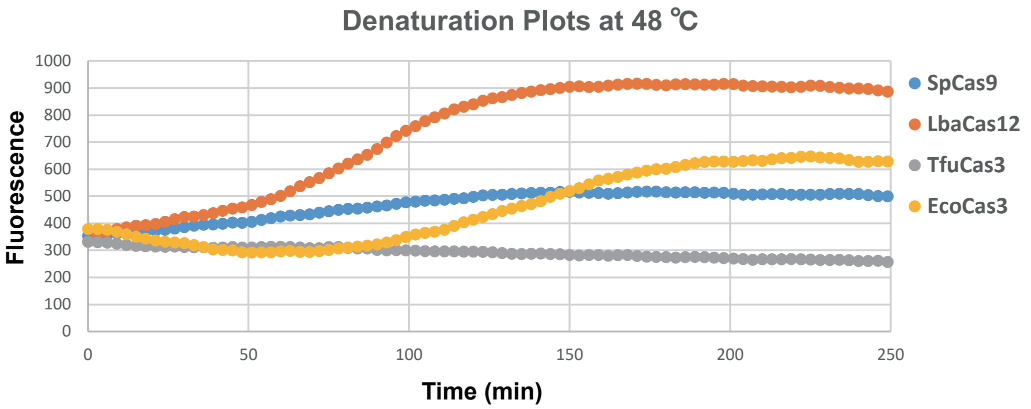

Supplementary Fig. 4. Temperature-dependent stability of recombinant EcoCas3 protein. We evaluated temperature-dependent stability using ProteoStat detection reagent (Enzo Life Sciences), which allowed the aggregation onset temperature to be determined. Vertical and horizontal axes show fluorescence intensity, which is dependent on protein denaturation, and time, respectively. Considering thermal stability at a constant temperature of 37°C, EcoCas3 was mostly denatured in 8 h, while SpCas9, LbaCas12, and TfuCas3 were not denatured after 24 h (upper). At a constant temperature of 48°C, LbaCas12a and SpCas9 proteins showed earlier aggregation onset than EcoCas3 (lower).

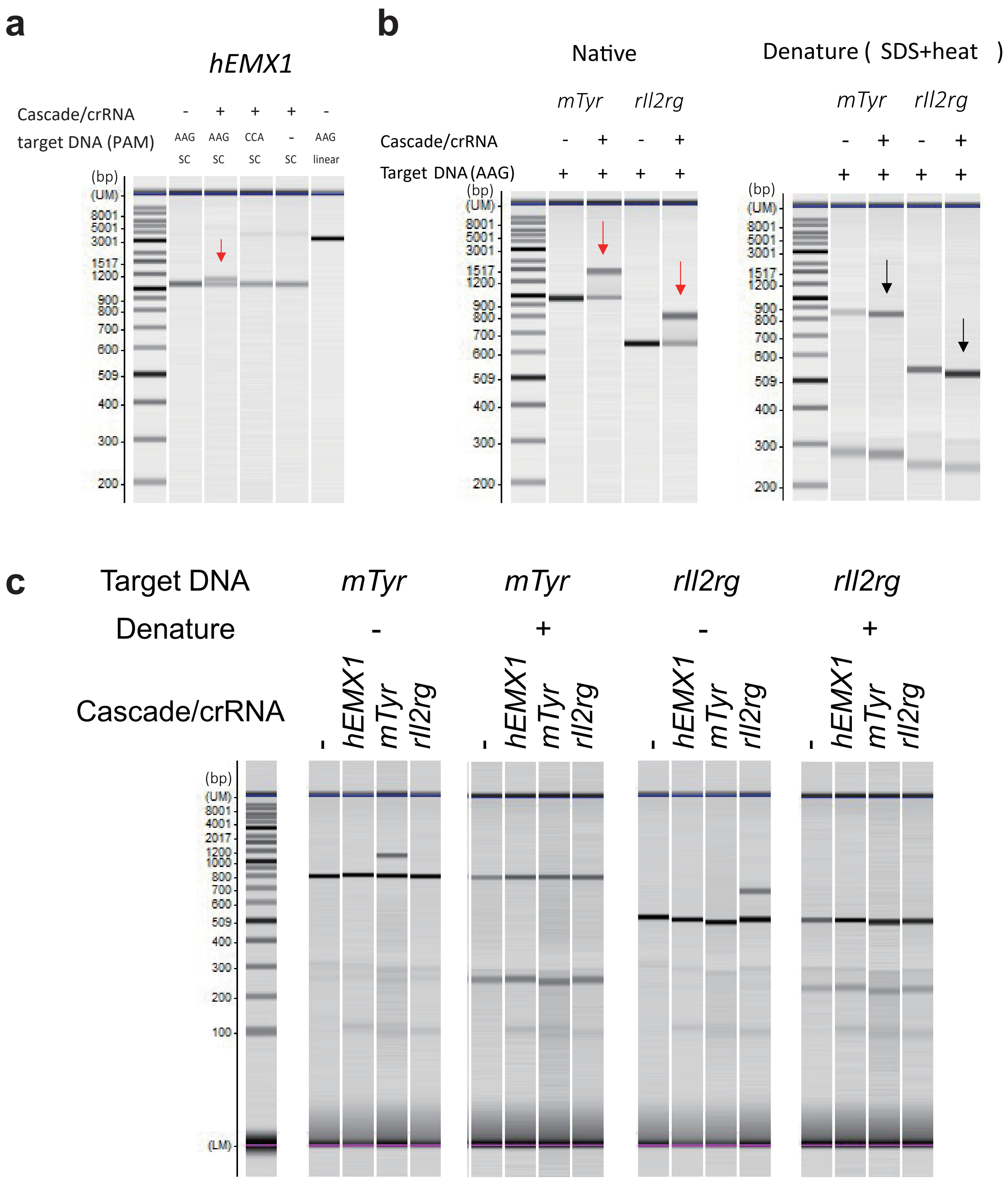

Supplementary Fig. 5. Evaluation of R-loop formation by electrophoretic mobility shift assay (EMSAs). (a) EcoCascade/crRNA complex binds to supercoiled (SC) plasmids containing *hEMX1* spacer sequences flanked by a PAM (AAG) as shown by a red arrow, but did not bind to a nonPAM (CCA). (b) The Cascade complex binding to the PCR products of other target sequences (*mTyr* and *rIl2rg* genes). Denaturation of the Cascade complex (0.08% SDS, 95°C, 2 min) abolished DNA binding (black arrows). (c) Exchange of nucleotide pairs between crRNAs and target sequences abolished the binding, indicating the specificity of target recognition by the EcoCascade/crRNA complex. These experiments are reproducible as they showed similar results with different targeted locus.

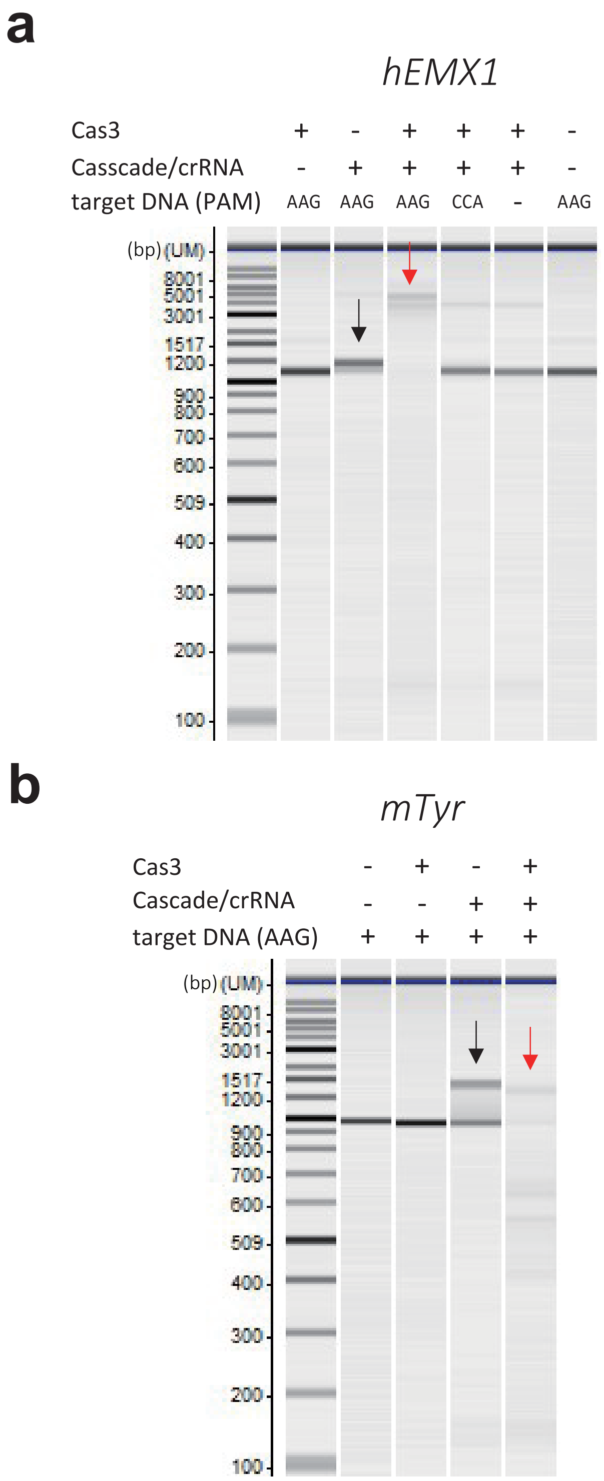

Supplementary Fig. 6. Electrophoretic mobility shift assay (EMSA) and DNA degradation assay. (a) EcoCascade/crRNA complex binds to target plasmids containing *hEMX1* spacer sequences flanked by PAM (AAG) (black arrow). EcoCas3 recruited into EcoCascade degrades the target DNA (red arrow). (b) EcoCascade binds to PCR products of the target DNA (*mTyr*) (black arrow), which is degraded by EcoCas3 (red arrow). This experiment is reproducible as a and b showed similar results with different targeted locus.

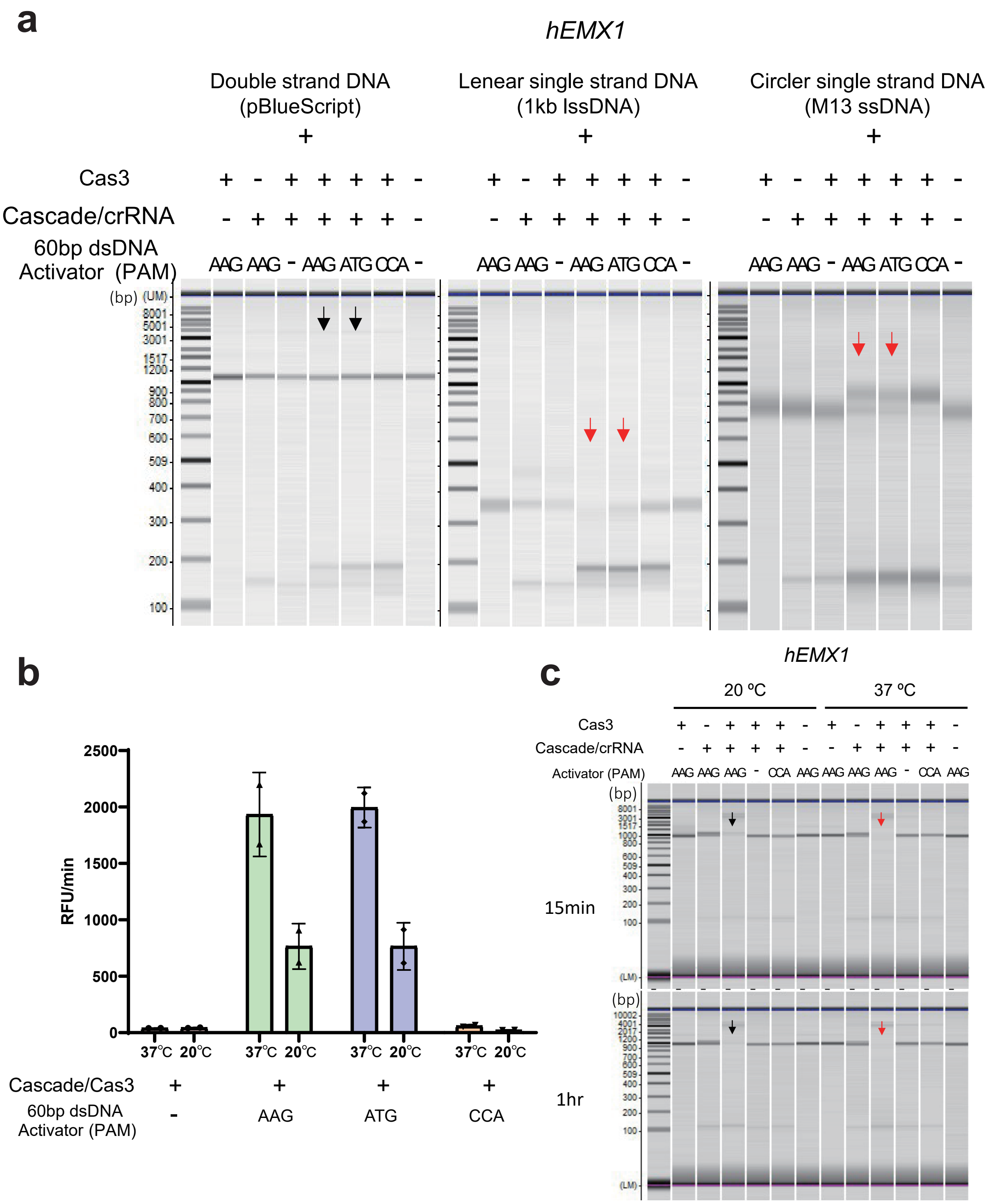

Supplementary Fig. 7. Collateral cleavages of nearby non-specific ssDNAs. (a) EcoCas3 possesses collateral non-specific single-stranded DNA (ssDNA) cleavage activity after target-specific double-stranded DNA (dsDNA) cleavage. Circular ssDNA M13 phage and linearized long ssDNA were degraded after incubation for 1 h at 37°C (red arrows), but circular pBlueScript dsDNA was not cleaved (black arrows). (b) Comparison of collateral cleavage activity between 37°C and 20°C. (c) *In vitro* reconstitution assay for target DNA degradation also showed higher activity at 37°C (red arrows) than at 20°C (black arrows). This experiment was repeated twice independently with similar results. Data are presented for n=2 independent measurements and mean value, error bars represent SD values.

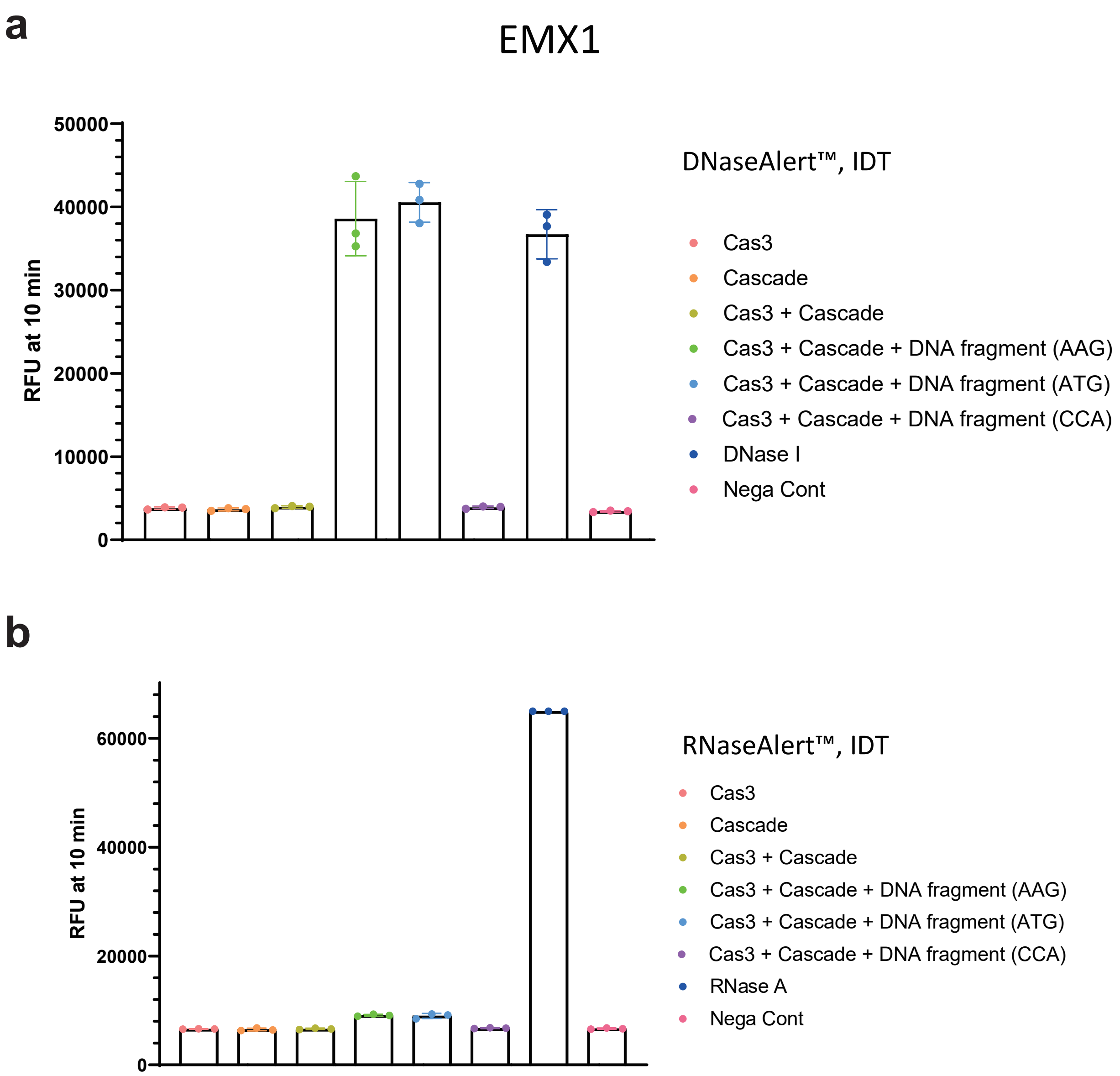

Supplementary Fig. 8. Collateral cleavage activity for ssDNAs and ssRNAs. (a) Using fluorescent reporter DNA oligonucleotides (DNaseAlert™, IDT), we detected collateral ssDNA cleavage activity by assembling EcoCas3, EcoCascade RNPs and dsDNA fragments that included target sequences (*hEMX1* or *mTyr*) flanked by PAM-AAG or -ATG, but not with PAM-CCA. (b) Little or no RNase activity was detected with fluorescent reporter RNA oligonucleotides (RNaseAlert™, IDT) by assembling EcoCas3, EcoCascade RNPs and dsDNA fragments. Data are presented for n=3 independent measurements and mean value, error bars represent SD values.

a

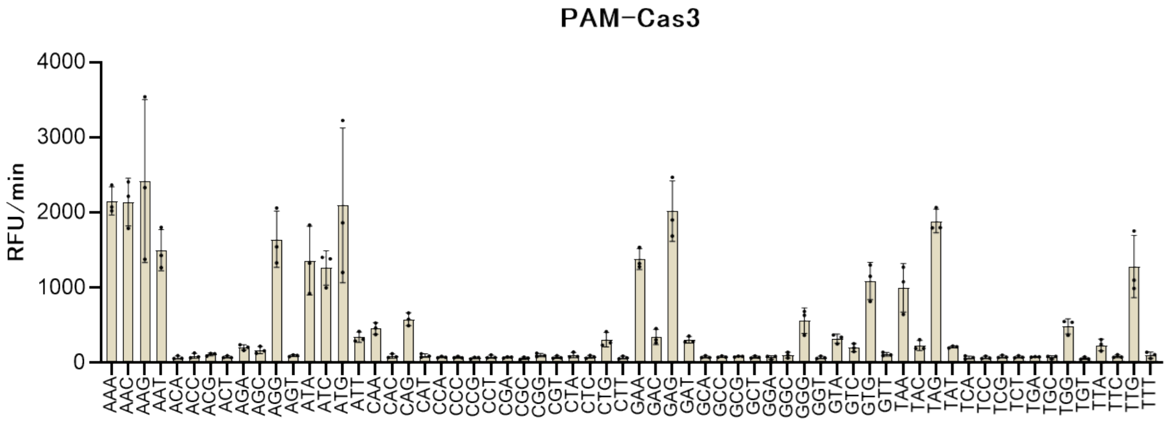

b

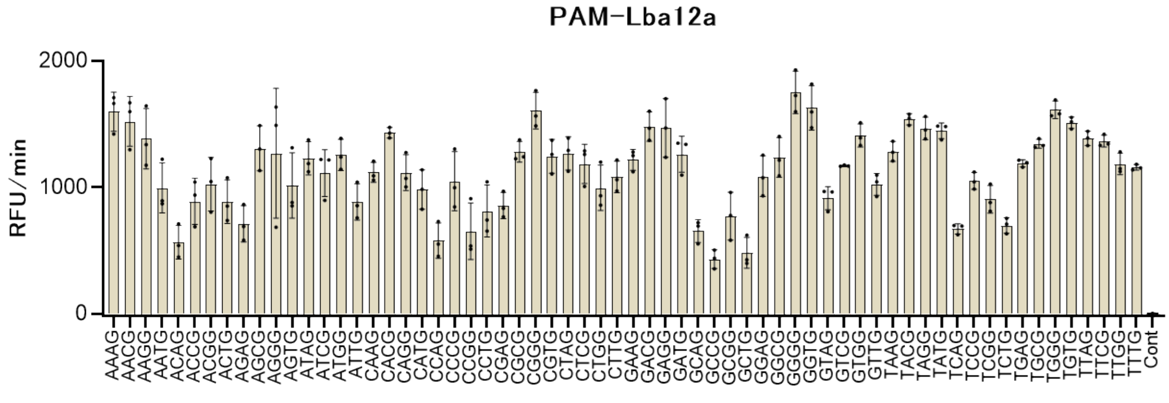

Supplementary Fig. 9. Specific PAM recognition for collateral ssDNA cleavage by EcoCas3. (a,b) Screening of all 64 possible target sites containing each of the three-nucleotide PAM sequences for *trans* cleavage activity by EcoCas3 (a) and LbaCas12a (b). Data are presented for n=3 independent measurements and mean value, error bars represent SD values.

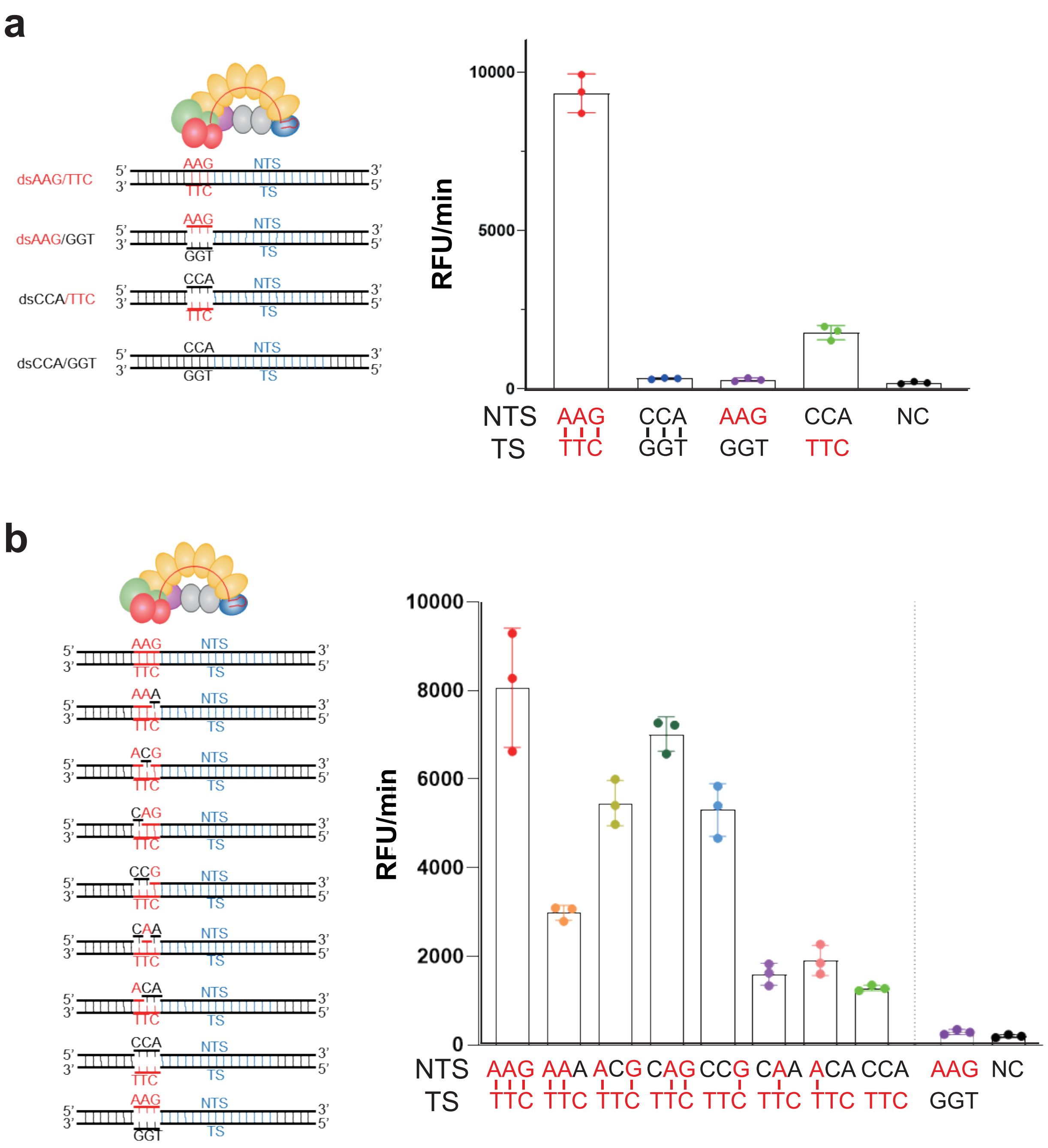

Supplementary Fig. 10. Collateral cleavage activity. (a) Collateral cleavage activated by dsDNA containing an unpaired PAM. (b) Screening of PAM base-pairing between every three PAM nucleotides for trans cleavage activity. Data are presented for n=3 independent measurements and mean value, error bars represent SD values.

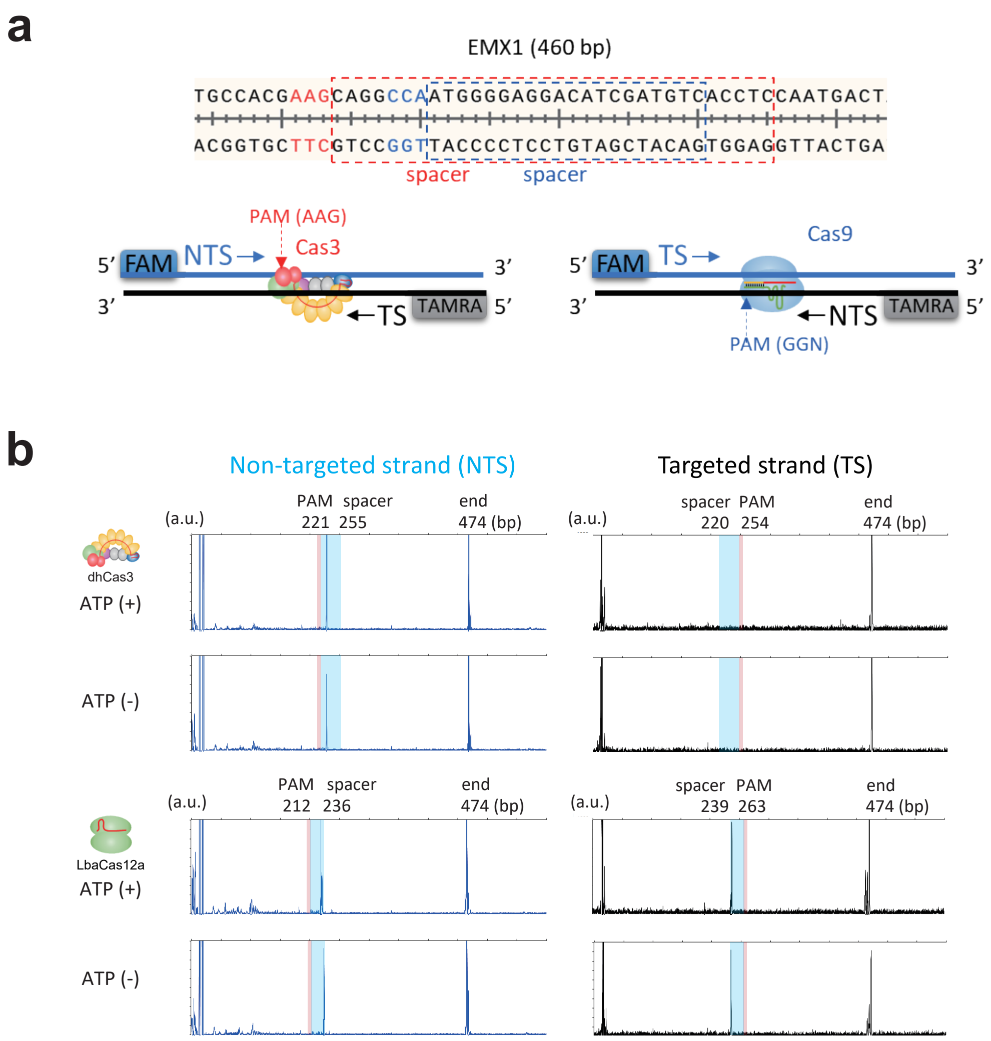

Supplementary Fig. 11. The dsDNA cleavage assay. (a) Schematic depictions of the dsDNA cleavage assay. Fluorescently-labeled target dsDNA substrates, 5′-NTS-FAM and 5′-TS-TAMRA, to visualize dsDNA cleavage. (b) The dhCas3 SF2 domain mutant cleaves the NTS in *cis*, but not the TS in *trans* in ATP (+ and -) reaction buffer, while LbaCas12a cleaves both the NTS and TS in ATP (+ and -) reaction buffer. The x-axis represents the DNA fragment size in base pairs (bp), and the y-axis represents the arbitrary unit (a.u.).

a

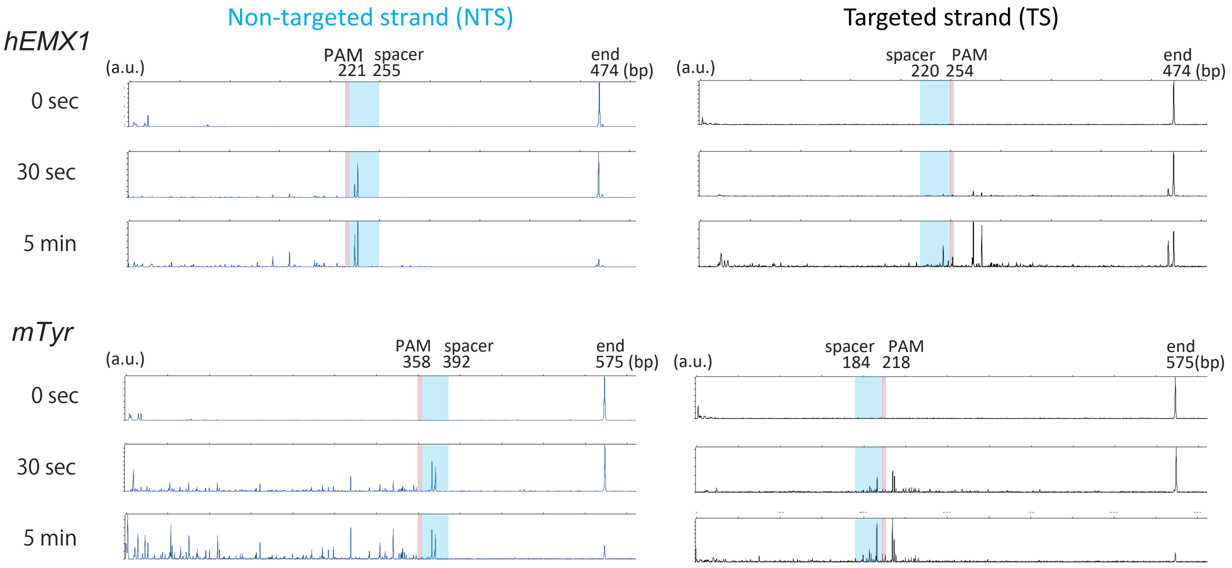

b

Sample File Name	Dye	Sample Peak	Size	Height
EMX1-Cas3 37-30sec.fsa	B	29	143.2	482
EMX1-Cas3 37-30sec.fsa	B	31	159.7	661
EMX1-Cas3 37-30sec.fsa	B	42	224.9	2391
EMX1-Cas3 37-30sec.fsa	B	43	228.0	6101
EMX1-Cas3 37-30sec.fsa	B	45	468.8	8146
EMX1-Cas3 37-30sec.fsa	Y	67	272.3	1103
EMX1-Cas3 37-30sec.fsa	Y	70	280.6	700
EMX1-Cas3 37-30sec.fsa	Y	130	467.0	1464
EMX1-Cas3 37-30sec.fsa	Y	133	472.3	8801
EMX1-Cas3 37-5min.fsa	B	33	143.1	810
EMX1-Cas3 37-5min.fsa	B	35	159.7	1172
EMX1-Cas3 37-5min.fsa	B	39	184.8	519
EMX1-Cas3 37-5min.fsa	B	51	225.1	2510
EMX1-Cas3 37-5min.fsa	B	52	228.1	3525
EMX1-Cas3 37-5min.fsa	B	57	468.9	619
EMX1-Cas3 37-5min.fsa	Y	16	20.5	728
EMX1-Cas3 37-5min.fsa	Y	87	242.2	1145
EMX1-Cas3 37-5min.fsa	Y	92	251.6	527
EMX1-Cas3 37-5min.fsa	Y	101	271.3	491
EMX1-Cas3 37-5min.fsa	Y	102	272.4	2415
EMX1-Cas3 37-5min.fsa	Y	106	280.8	2203
EMX1-Cas3 37-5min.fsa	Y	167	467.0	1393
EMX1-Cas3 37-5min.fsa	Y	170	472.4	1901
EMX1-Cas9 37-5min.fsa	B	6	229.2	15251
EMX1-Cas9 37-5min.fsa	B	9	468.9	10292
EMX1-Cas9 37-5min.fsa	Y	55	229.0	593
EMX1-Cas9 37-5min.fsa	Y	57	236.2	583
EMX1-Cas9 37-5min.fsa	Y	58	237.0	1500
EMX1-Cas9 37-5min.fsa	Y	59	238.0	7933
EMX1-Cas9 37-5min.fsa	Y	109	468.8	472
EMX1-Cas9 37-5min.fsa	Y	110	472.4	9178
EMX1-control.fsa	B	8	469.0	4667
EMX1-control.fsa	Y	118	472.5	5050
Tyr-Cas3 37-30sec.fsa	B	16	52.5	401
Tyr-Cas3 37-30sec.fsa	B	22	75.0	401
Tyr-Cas3 37-30sec.fsa	B	28	109.4	406
Tyr-Cas3 37-30sec.fsa	B	56	269.1	699
Tyr-Cas3 37-30sec.fsa	B	63	320.2	487
Tyr-Cas3 37-30sec.fsa	B	75	366.3	1410
Tyr-Cas3 37-30sec.fsa	B	77	370.5	1148
Tyr-Cas3 37-30sec.fsa	B	79	573.3	2171
Tyr-Cas3 37-30sec.fsa	Y	83	215.7	914
Tyr-Cas3 37-30sec.fsa	Y	91	234.2	1258
Tyr-Cas3 37-30sec.fsa	Y	204	573.2	2608
Tyr-Cas3 37-5min.fsa	B	14	53.5	610
Tyr-Cas3 37-5min.fsa	B	20	74.4	447
Tyr-Cas3 37-5min.fsa	B	21	75.2	544
Tyr-Cas3 37-5min.fsa	B	29	109.4	536
Tyr-Cas3 37-5min.fsa	B	53	269.0	585
Tyr-Cas3 37-5min.fsa	B	61	320.2	436
Tyr-Cas3 37-5min.fsa	B	70	366.3	456
Tyr-Cas3 37-5min.fsa	Y	85	215.6	1099
Tyr-Cas3 37-5min.fsa	Y	93	234.3	987
Tyr-Cas3 37-5min.fsa	Y	94	236.4	514
Tyr-Cas3 37-5min+h.fsa	B	8	22.6	609
Tyr-Cas3 37-5min+h.fsa	B	9	25.9	408
Tyr-Cas3 37-5min+h.fsa	B	16	53.6	873
Tyr-Cas3 37-5min+h.fsa	B	23	75.1	594
Tyr-Cas3 37-5min+h.fsa	B	31	109.5	592
Tyr-Cas3 37-5min+h.fsa	B	41	160.4	425
Tyr-Cas3 37-5min+h.fsa	B	60	269.1	806
Tyr-Cas3 37-5min+h.fsa	B	65	304.2	424
Tyr-Cas3 37-5min+h.fsa	B	70	320.1	780
Tyr-Cas3 37-5min+h.fsa	B	82	366.3	754
Tyr-Cas3 37-5min+h.fsa	B	84	370.5	631
Tyr-Cas3 37-5min+h.fsa	Y	78	206.7	624
Tyr-Cas3 37-5min+h.fsa	Y	79	208.8	403
Tyr-Cas3 37-5min+h.fsa	Y	84	215.6	1898
Tyr-Cas3 37-5min+h.fsa	Y	94	234.3	2154
Tyr-Cas3 37-5min+h.fsa	Y	95	236.4	1147
Tyr-Cas3 37-5min+h.fsa	Y	96	238.4	417
Tyr-Cas3 37-5min+h.fsa	Y	183	573.1	486
Tyr-control.fsa	B	7	573.3	3493
Tyr-control.fsa	Y	148	573.2	3565

Supplementary Fig. 12. The dsDNA cleavage assay. (a) Comparison of 30 sec (short) and 5 min (long) incubation for the dsDNA cleavage assay (*hEMX1* or *mTyr*). The x-axis represents the DNA fragment size in base pairs (bp), and the y-axis represents the arbitrary unit (a.u.). (b) The size and patterns of cleaved fragments after short and long incubation in the dsDNA cleavage assay.

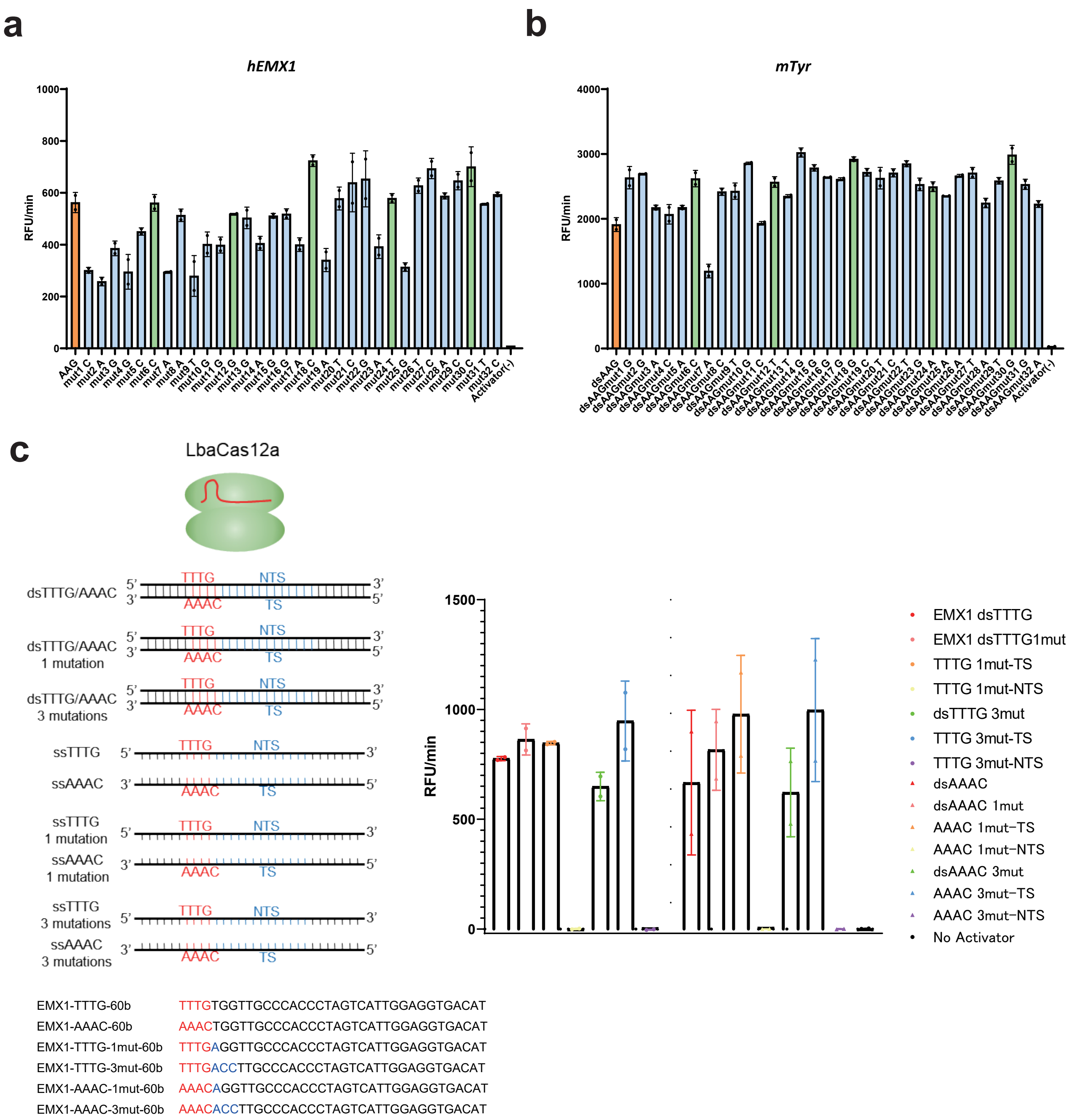

Supplementary Fig. 13. The effect of mismatch of each 32-nt spacer sequence on collateral cleavage activity. (a,b) A single mismatch in the spacer region has a little or no effect on collateral cleavage activity of *hEMX1* target (a) and *mTyr* target (b). (c) One to three mismatches in the PAM sites and the spacer region have little effect on collateral cleavage activity by LbaCas12a. Data are presented for n=2 independent measurements and mean value, error bars represent SD values.

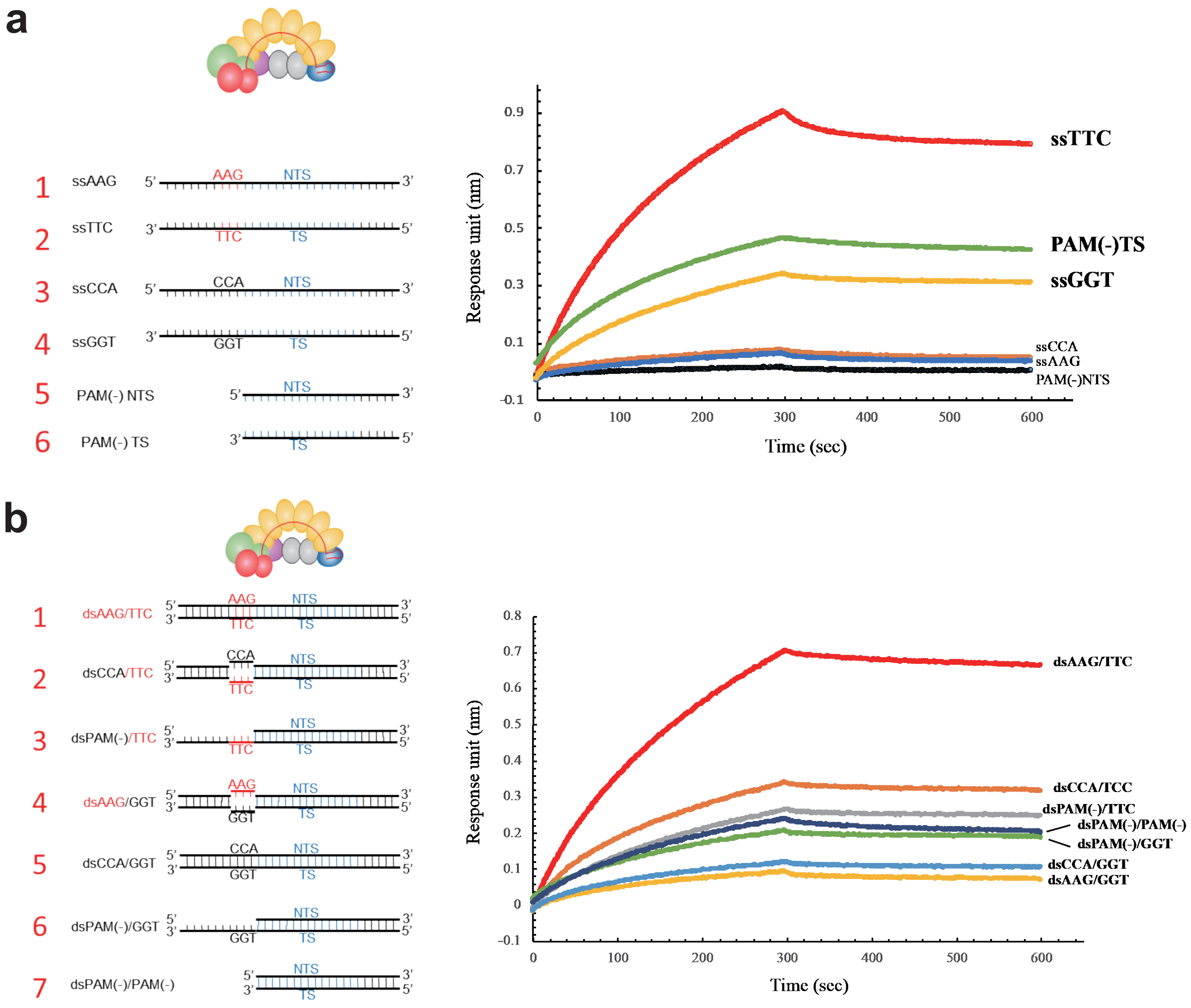

Supplementary Fig. 14. EcoCascade-target DNA associations and dissociations measured by the Octet RED 96 System. (a) crRNA-complementary ssDNA with TS-PAM (TTC) showing collateral cleavage (Fig. 2c) represents higher association than TS-nonPAM or TS-PAMless. (b) The interactions between Cascade and dsDNAs containing paired PAM (AAG-TTC), unpaired PAM between TS-PAM (TTC) and NTS-nonPAM (CCA), and unpaired PAM between NTS-PAM (AAG) and TS-nonPAM (GGT), correspond with the results from the collateral cleavage assay (Fig. 2f).

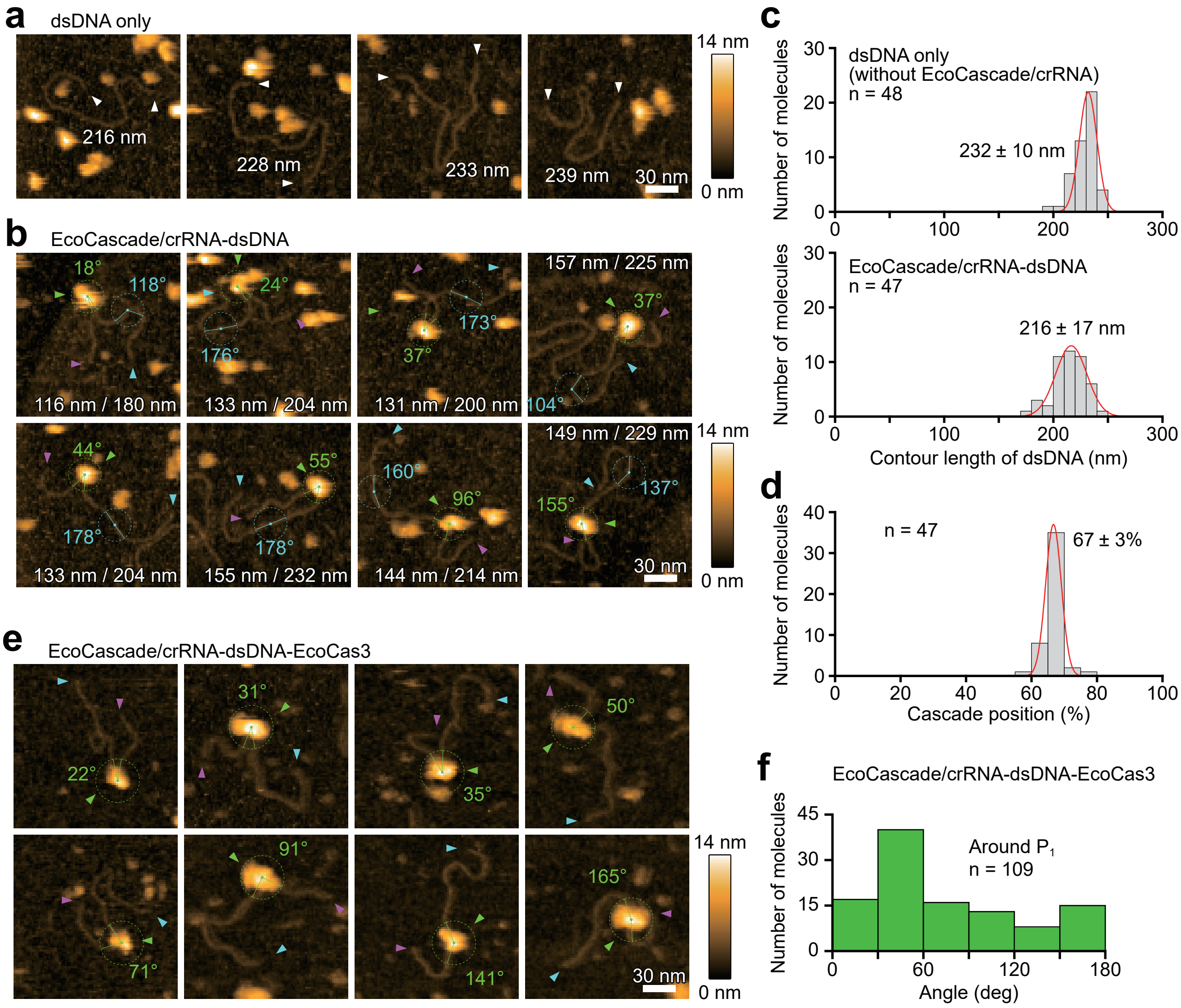

Supplementary Fig. 15. Quantitative characterization of Cascade-DNA binding. (a) Typical images of DNA without Cascade. Measured DNA length indicated by white letters. DNA ends: white arrowheads. Scan area: 150 × 150 nm^2 with 80 × 80 pixels; frame time: 0.2 s (line rate: 400 Hz). (b) Typical images of EcoCascade-dsDNA bindings. The short DNA from the cascade: magenta arrowheads, The long DNA from the cascade: light blue arrowheads. Cascade binding site: green arrowheads. Measured DNA length indicated by white letters. The angle of the DNA with and without Cascade is also shown in green and blue, respectively. (c) Histograms of DNA length with Cascade (bottom) and without Cascade (top). Mean ± S.D. (d) Histogram of the ratio of the length of long DNA to total length. Mean ± S.D. (e) Typical images of EcoCas3-EcoCascade-dsDNA complexes. R:20 nm. (f) Histograms of DNA angles around P1 in EcoCas3-EcoCascade-dsDNA. The each experiments in a, b, e were repeated twice independently with similar results.

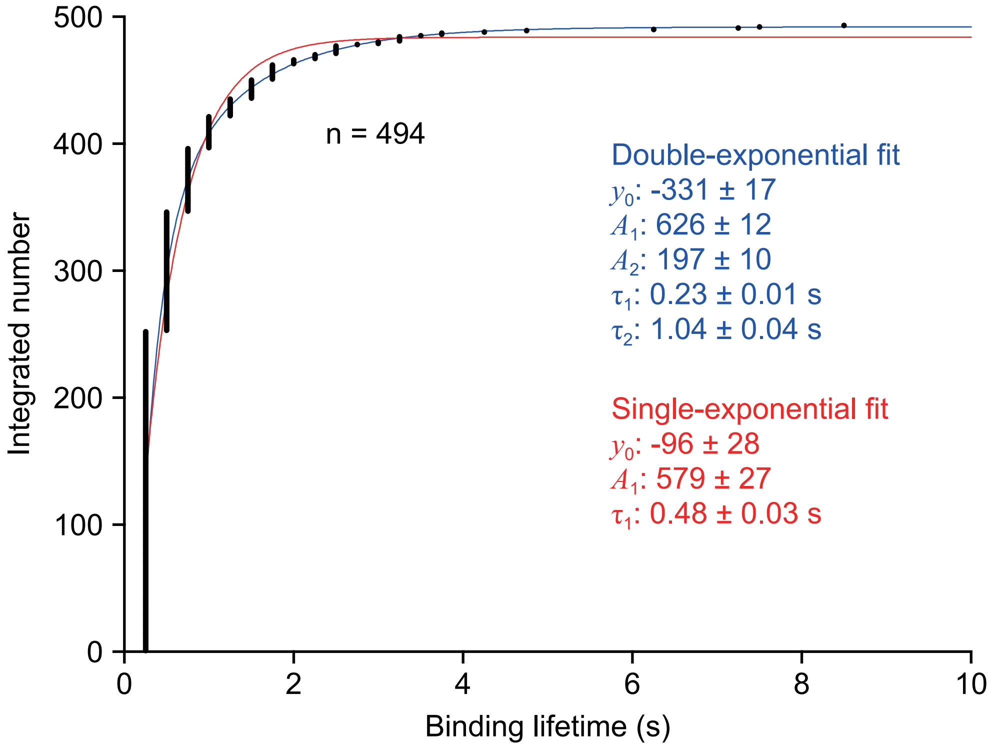

Supplementary Fig. 16. Integral analysis of the lifetime of non-specific bindings of a Cascade complex to DNA sites. Blue and red lines are fitted results for double and single exponential, respectively. The double exponential was statistically fitted (*one tailed* F-test, $P = 1.13\times10^{-12}$).

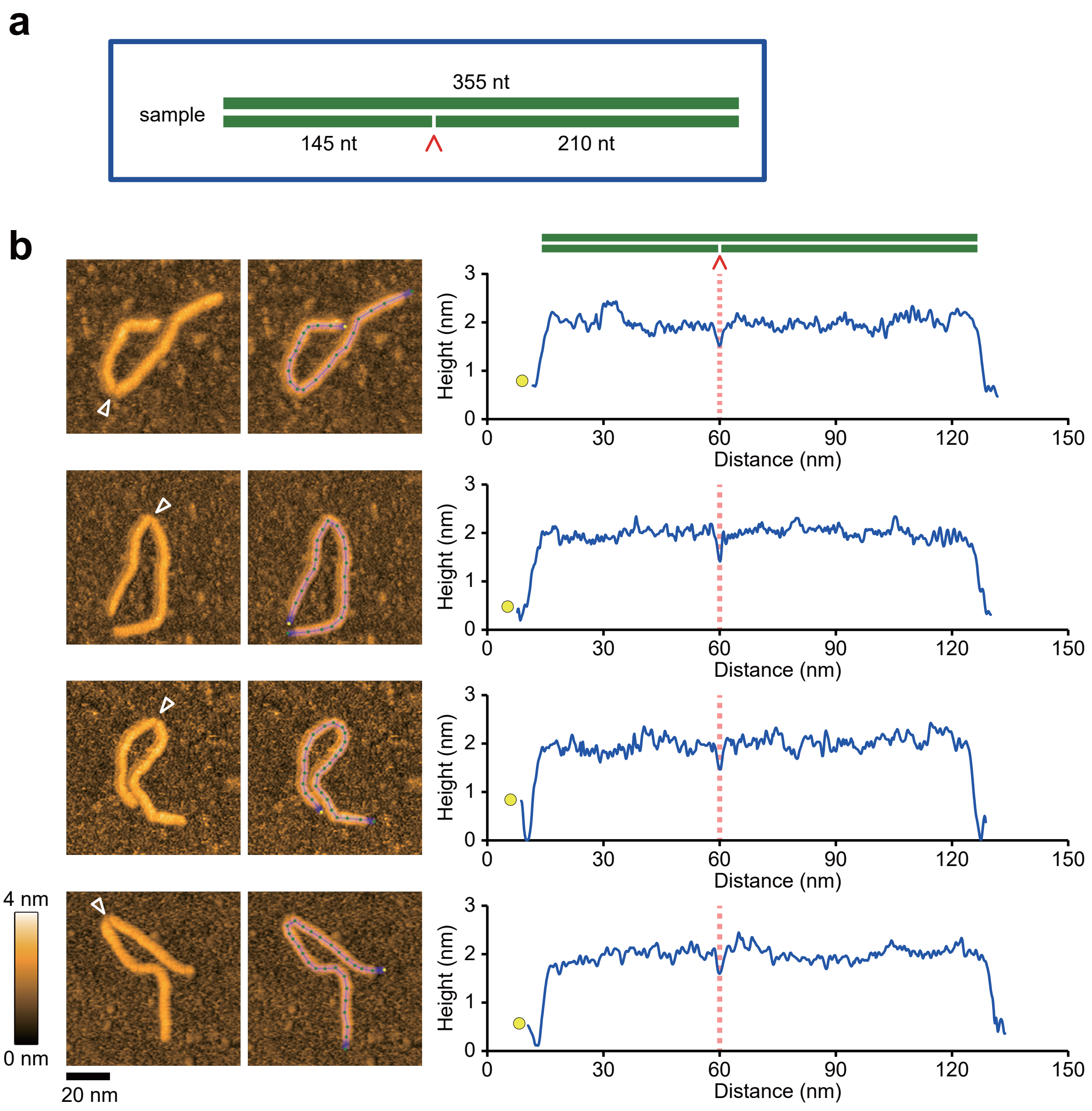

Supplementary Fig. 17. Visualization and characterization of artificial nicking. (a) The diagram of dsDNA which was artificially nicked by Nb.BsrDI endonuclease (red arrowhead). (b) Typical AFM images showing the nicked dsDNA (left and center) and the cross-sections along DNA ridge (right). The white arrowheads in the left images indicate the decreasing height at nicked site. The blue lines in the center images were drawn along the DNA ridges from the yellow dots, along which the cross-sections were measured. The representative images in b were collected from repeated two experiments independently with similar results.

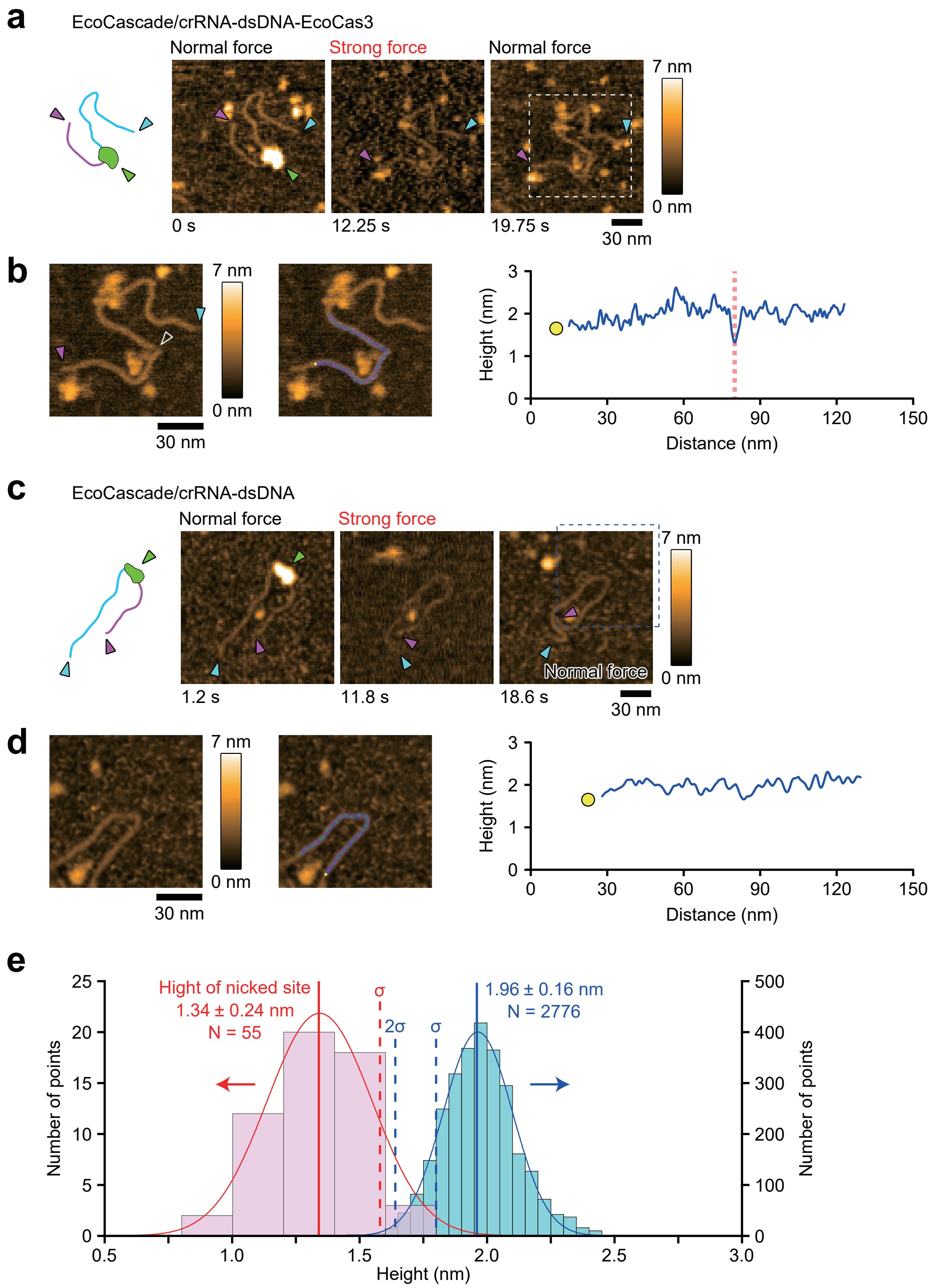

Supplementary Fig. 18. Characterization of Cas3-mediated nicking at no-ATP condition. (a) AFM images before and after removal of EcoCas3-EcoCascade-dsDNA by strong tapping force in the absence of ATP. Scan area: 150 × 150 nm^2 with 80 × 80 pixels; frame time: 0.25 s (line rate: 320 Hz). (b) Enlarged image of white dashed line in (a) and height measurement of the dsDNA. Scan area: 100 × 100 nm^2 with 200 × 200 pixels; frame time: 0.33 s (line rate: 606 Hz). Nick position: red dashed line. (c) AFM images before and after removal of EcoCascade-dsDNA by strong tapping force. Scan area: 150 × 150 nm^2 with 80 × 80 pixels; frame time: 0.2 s (line rate: 400 Hz). (d) Enlarged image of blue dashed line in (c) and height measurement of the dsDNA. Scan area: 100 × 100 nm^2 with 80 × 80 pixels; frame time: 0.2 s (line rate: 400 Hz). (b, d) The cross-section (right graph) was measured along the blue line drawn in the second image from left. The representative images in a-d were collected from repeated two experiments independently with similar results. (e) Distribution of measured DNA heights of the nicked (n=55) and another sites (n=2776). The mean heights and standard deviations (σ) are shown in the graph.

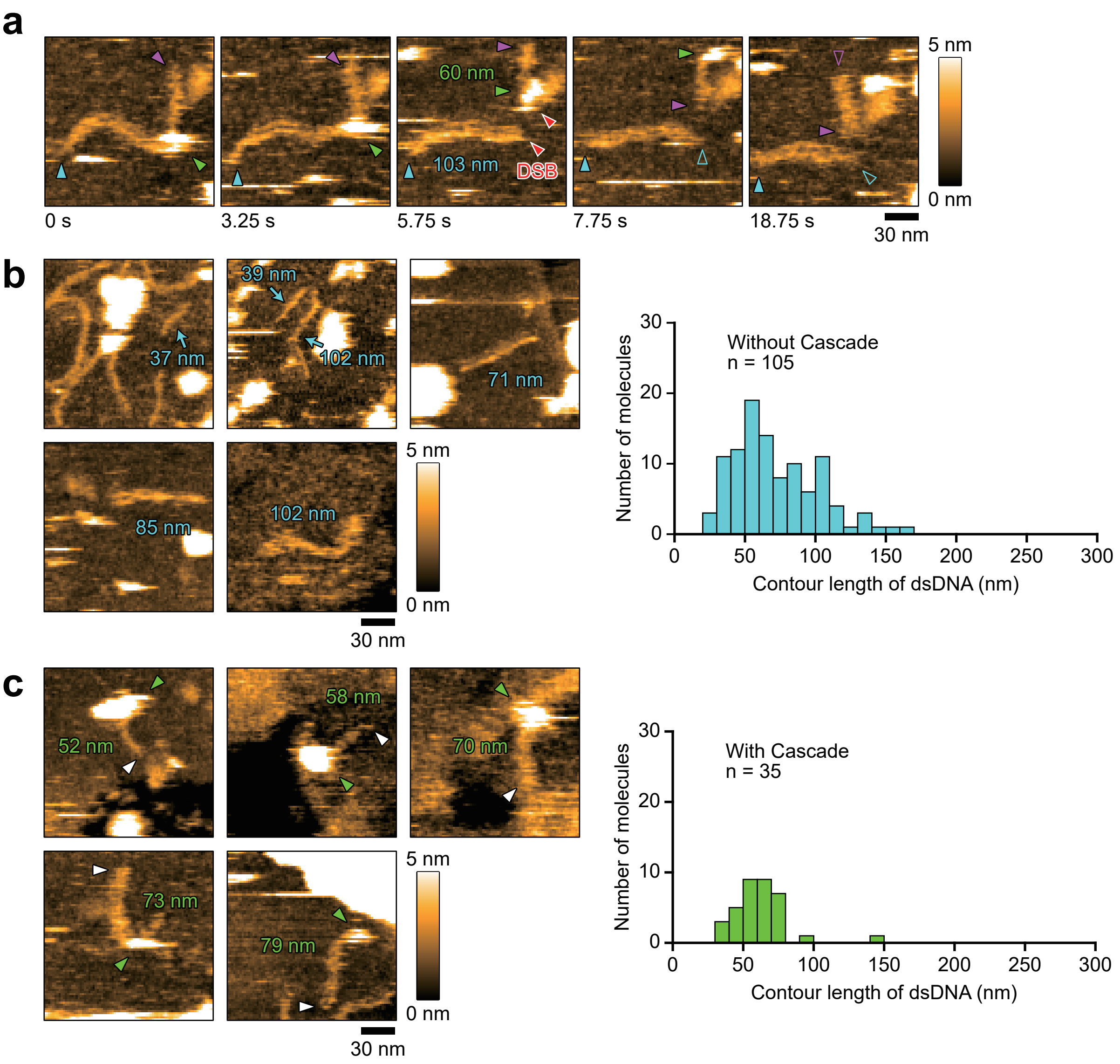

Supplementary Fig. 19. DNA length observed after adding Cas3 and ATP. (a) Typical images of a double strand break (DSB) by CRISPR-Cas3. The DSB was observed at 5.75 sec. A protein complex remained on the short DNA at 7.75 sec, but was dissociated at 18.75 sec. Scan area: 150 × 150 nm^2 with 80 × 80 pixels; frame time: 0.25 s (line rate: 320 Hz). (b) A typical AFM image of the fragmented DNA without proteins and a histogram of the DNA length observed after Cas3 reaction with ATP. (c) A typical image of the fragmented DNA with proteins and a histogram of the DNA length observed after Cas3 reaction with ATP. The experiment was repeated twice independently with similar results and collected individual data for quantification from both experiments.

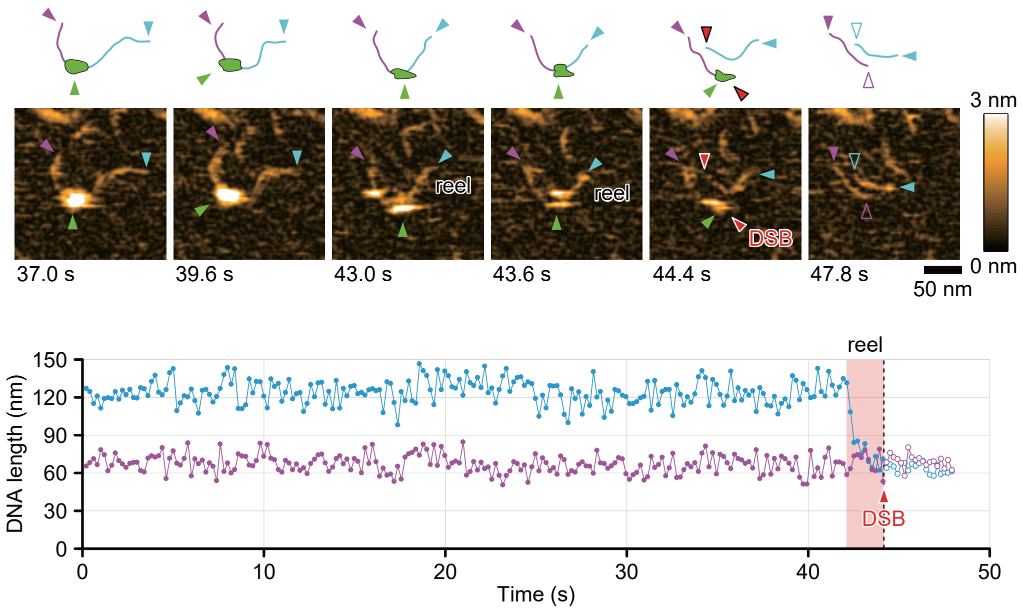

Supplementary Fig. 20. Dynamic visualization of CRISPR interference by hs-AFM.

In ATP (+) reaction buffer, the EcoCas3-Cascade complex repeatedly reels and releases the longer side of the DNA (blue arrows) and then cleaves it with a DSB (red arrows) (video 5). Scan area: 150 × 150 nm² with 80 × 80 pixels; frame time: 0.2 s (line rate: 400 Hz). This similar data were collected from an independently replicated experiment in Figure 5b.

Supplementary Table 1. Binding kinetics between EcoCascade and ligands.

		Response unit	Rmax	*k* on (1/Ms)	*k* dis (1/s)	KD (M)	χ^2	R^2
**Supplementary Figure 14a**								
2	ssTTC	0.8909	1.0763	3.08E+05	4.84E-04	1.57E-09	0.021167	0.9991
4	ssGGT	0.3319	0.4261	2.53E+05	2.96E-04	1.17E-09	0.004821	0.998807
6	dsPAM(-)TS	0.4566	0.4334	5.27E+05	3.60E-04	6.83E-10	0.020497	0.996236
**Supplementary Figure 14b**								
1	dsAAG/TCC	0.6917	0.8122	2.94E+05	1.88E-04	6.40E-10	0.002915	0.999833
2	dsCCA/TCC	0.3324	0.3469	3.86E+05	1.90E-04	4.93E-10	0.002682	0.999266
3	dsPAM(-)/TTC	0.2587	0.2976	3.04E+05	1.67E-04	5.50E-10	0.001006	0.999572
4	dsAAG/GGT	0.0902	0.1259	2.42E+05	5.83E-04	2.41E-09	0.002105	0.990738
5	dsCCA/GGT	0.116	0.1377	3.11E+05	3.42E-04	1.10E-09	0.00186	0.99575
6	dsPAM(-)/GGT	0.2026	0.1828	5.57E+05	1.75E-04	3.14E-10	0.008907	0.991191
7	dsPAM(-)/PAM(-)	0.2344	0.2585	3.53E+05	4.83E-04	1.37E-09	0.002376	0.998416
**Figure 3e**								
	n0	0.0132	n.c.	n.c.	n.c.	n.c.	n.c.	n.c.
	n6	0.0857	0.0929	2.15E+06	1.07E-02	4.99E-09	0.00041	0.995655
	n12	0.1117	0.1096	1.10E+06	2.71E-03	2.47E-09	0.000333	0.993216
	n18	0.3791	0.3619	6.41E+05	6.96E-04	1.09E-09	0.00034	0.999753
	n24	0.4115	0.3558	8.43E+05	8.19E-04	9.72E-10	0.001636	0.998749
	n30	0.3699	0.3453	7.31E+05	6.92E-04	9.47E-10	0.000465	0.999709
	n32	0.6482	0.4785	6.21E+05	2.21E-04	3.56E-10	0.002651	0.99965

k on ($M^{-1}s^{-1}$) showing a binding rate constant, respectively. n.c. mean Not Calculated.

Supplementary Table 2. Target sequences for Type 1-E CRISPR, CRISPR-Cas12a and CRISPR-Cas9

Name	PAM (5' to 3')	Sequence (5' to 3')
Eco CRISPR-Cas3		
EMX1-AAG	AAG	CAGGCCAATGGGGAGGACATCGATGTCACCTC
mTyr-AAG	AAG	GGACACACTGCTTGGGGGCTCTGAAATATGGA
rIl2rg-AAG	AAG	ATATCCAGCTCTACCAGACATTTGTTGTCCAG
LbaCas12a		
EMX1-TTTG	TTTG	TGGTTGCCCACCCTAGTCATT
Tyr-TTTG	TTTG	TATGGATGCATTACTATGTGT
SpCas9		
EMX1	CCA	ATGGGGAGGACATCGATGTC

Supplementary Table 3. Primers used for cloning and genotyping.

Name	Sequence (5' to 3')	Note
GAPDH Exon Location2-3	5HEX/AAGGTCGGA/ZEN/GTCAACGGATTTGGTC/3IABkFQ	[illegible] assay
EMX1-400bp-F	CTTTGCTGGGGCTAGAGGAG	Preparation of donor DNAs for AFM
EMX1-small-R1	GAGGAGAAGGCCAAGTGGTC	
EMX1-FA-FAM-500bp-F	FAM/CCATCAGGCTCTCAGCTCAG	Preparation of donor DNAs for fragment analysis
EMX1-FA-TAMRA-500bp-R	TAMRA/GAGGAGAAGGCCAAGTGGTC	
Tyr-FA-FAM-600bp-F	FAM/TGGACCTCAGTTCCCCTTCA	
Tyr-FA-TAMRA-600bp-R	TAMRA/GGTGACGACCTCCCAAGTAC	
mTyr-F1	AGAACTTGTTGGCAAAAGAATGCTG	Preparation of donor DNAs for electrophoretic mobility shift assay
mTyr-R1	TGAAAATGTGGCTGCTGAAG	
rIl2rg-ex3-F1	GGGCAGATGTGGTACCTACC	
rIl2rg-ex3-R1	TCTGCTTTTCCACCTCAGTTCT	
