# Supplementary material for: Dynamic mechanisms of CRISPR interference by Escherichia coli CRISPR-Cas3
Source: Nat Commun. 2022 Aug 30;13:4917. doi: 10.1038/s41467-022-32618-0 (PMC9427990; doi:10.1038/s41467-022-32618-0)
Supplement: Supplementary file 3 — Description of Additional Supplementary Files [file 41467_2022_32618_MOESM3_ESM.pdf]

**Description of Additional Supplementary Files**

File Name: Supplementary Movie 1
Description: EcoCascade RNP searches for the right PAM site from one end to the other through the target DNA (Fig. 4b). Z-scales of left and right videos are 0– 14 nm and 0–3.6 nm, respectively. Scan area: 200 × 200 nm2 with 80 × 80 pixels; frame time: 0.25 s (line rate: 320 Hz). To remove the high-frequency noise and the surface tilt, all videos are treated with an intraframe Gaussiankernel filter with the standard deviation of 0.68 pixel and a firstorder flatten filter in both the XY-axes, respectively. For the right video, to improve the visibility of the molecules of interest, the low-frequency surface undulation due to the hetero lipid membrane surface was further removed by the “rolling ball” background subtraction 73 with the ball diameter of 34.5 nm. This image processing was also applied to video 2 and the right ones of videos 5-7.

File Name: Supplementary Movie 2
Description: Three typical videos showing EcoCascade RNP binding to the target site of the dsDNA to form R-loop architecture. Scan area: 200 × 200 nm2 with 80 × 80 pixels; frame time: 0.25 s (line rate: 320 Hz); Z-scales: 0–3.6 nm. The green open and closed triangles indicate the EcoCascade RNP searching for the target site and the EcoCascade RNP bound to the target site, respectively.

File Name: Supplementary Movie 3
Description: EcoCas3 combined with EcoCascade RNPs mediates nicking at the target site of the 600-bp DNA in ATP-free (-) reaction buffer (Fig. 4c). Scan area: 150 × 150 nm2 (first half) and 100 × 100 nm2 (second half) with 80 × 80 pixels; frame time: 0.2 s (line rate: 400 Hz). Zscales of the first half and the second half are 0–13 nm and 0–6 nm, respectively.

File Name: Supplementary Movie 4
Description: Extensive tapping forces remove EcoCas3-Cascade RNP from DNA, and a nicklike shape appears at the DNA site where the EcoCas3-Cascade RNP had bound (Supplementary Fig. 18a, b). Scanning condition of the first half, scan area: 150 × 150 nm2 with 80 × 80 pixels; frame time: 0.25 s (line rate: 320 Hz). Scanning condition of the second half, scan area: 100 × 100 nm2 with 200 × 200 pixels; frame time: 0.33 s (line rate: 606 Hz). Zscale: 0–7 nm. To improve visibility of the nick-like shape, the second half video was treated with an interframe triangular-kernel filter with the range of 2 frames.

File Name: Supplementary Movie 5
Description: EcoCas3-Cascade complex repeatedly reels and releases the longer side of the DNA, then cleaves it with a DSB in ATP (+) reaction buffer (Supplementary Fig. 20). Scan area: 150× 150 nm2 with 80×80 pixels; frame time: 0.2 s (line rate: 400 Hz). Z-scales of left and right videos are 0–14 nm and 0–3 nm, respectively. For the right video, to improve the visibility of the molecules of interest, the high-frequency noise was further removed by an interframe triangular-kernel filter with the range of 2 frames. This image processing was also applied to the right ones of video 6 and 7.

File Name: Supplementary Movie 6
Description: EcoCas3-Cascade complex reels the longer side of the DNA, then cleaves it with a DSB in ATP (+) reaction buffer (Fig. 4d). Scan area: 200 × 200 nm2 with 80 × 80 pixels; frame time: 0.2 s (line rate: 400 Hz). Z-scales of left and right videos are 0–14 nm and 0–3 nm, respectively.

File Name: Supplementary Movie 7
Description: EcoCas3-Cascade complex reels the longer side of the DNA, then cleaves it with a DSB in ATP (+) reaction buffer. Scan area: 200 × 200 nm2 with 80 × 80 pixels; frame time: 0.2 s (line rate: 400 Hz). Z-scales of left and right videos are 0–14 nm and 0–3 nm, respectively.

File Name: Supplementary Data 1
Description: Coding sequences cloned in the plasmids are listed. An EcoCas3 cDNA with a octa-histidine tag and a six asparagine-histidine repeat tag was cloned into a pFastbac-1 plasmid (Thermo Fisher Scientific, Waltham, Massachusetts, USA). EcoCas11 with a hexahistidine tag and HRV3C protease recognition site, EcoCascade operon, and pre-crRNA were cloned into pCDFDuet-1, pRSFDuet1, and pACYCDuet-1 plasmids, respectively (Supplementary Fig. 2c).

File Name: Supplementary Data 2
Description: For in vitro DNA cleavage assay, targeted sequences of EMX1 with PAM variants (5'-AAG-3' or 5'-CCA-3') were cloned into a pCR4Blunt-TOPO plasmid vector (Thermo Fisher Scientific). For collateral DNA cleavage assays, 60 bp activator fragments of hEMX1 and mTyr were designed for CRISPR-Cas3, CRISPR-Cas12a and CRISPR-Cas9.
