# Supplementary material for: Dynamic mechanisms of CRISPR interference by Escherichia coli CRISPR-Cas3
Source: Nat Commun. 2022 Aug 30;13:4917. doi: 10.1038/s41467-022-32618-0 (PMC9427990; doi:10.1038/s41467-022-32618-0)
Supplement: Supplementary file 13 — Reporting Summary [file 41467_2022_32618_MOESM13_ESM.pdf]

nature portfolio

Corresponding author(s): Tomoji Mashimo

Last updated by author(s): Jul 8, 2022

Reporting Summary

Nature Portfolio wishes to improve the reproducibility of the work that we publish. This form provides structure for consistency and transparency in reporting. For further information on Nature Portfolio policies, see our Editorial Policies and the Editorial Policy Checklist.

Statistics

For all statistical analyses, confirm that the following items are present in the figure legend, table legend, main text, or Methods section.

n/a	Confirmed	
☐	☒	The exact sample size (*n*) for each experimental group/condition, given as a discrete number and unit of measurement
☐	☒	A statement on whether measurements were taken from distinct samples or whether the same sample was measured repeatedly
☐	☒	The statistical test(s) used AND whether they are one- or two-sided<br>*Only common tests should be described solely by name; describe more complex techniques in the Methods section.*
☒	☐	A description of all covariates tested
☐	☒	A description of any assumptions or corrections, such as tests of normality and adjustment for multiple comparisons
☐	☒	A full description of the statistical parameters including central tendency (e.g. means) or other basic estimates (e.g. regression coefficient) AND variation (e.g. standard deviation) or associated estimates of uncertainty (e.g. confidence intervals)
☐	☒	For null hypothesis testing, the test statistic (e.g. *F*, *t*, *r*) with confidence intervals, effect sizes, degrees of freedom and *P* value noted<br>*Give P values as exact values whenever suitable.*
☒	☐	For Bayesian analysis, information on the choice of priors and Markov chain Monte Carlo settings
☒	☐	For hierarchical and complex designs, identification of the appropriate level for tests and full reporting of outcomes
☒	☐	Estimates of effect sizes (e.g. Cohen's *d*, Pearson's *r*), indicating how they were calculated

Our web collection on statistics for biologists contains articles on many of the points above.

Software and code

Policy information about availability of computer code

Data collection	Peakscanner 2.0 (https://www.thermofisher.com/order/catalog/product/4381867) was used.
Data analysis	GraphPad Prism 8 (version 8.4.3), Microsoft Excel (version 15.0) was used for the statistical analyses. Kodec was used for the AFM analyses. The original code for Kodec 4.4.7.39 is opened to the public at the following web site: https://elifesciences.org/content/4/e04806/article-data#fig-data-supplementary-material. ForteBio analysis software for Octet (Version 7.0.1.27) was used for Protein interaction analysis.

For manuscripts utilizing custom algorithms or software that are central to the research but not yet described in published literature, software must be made available to editors and reviewers. We strongly encourage code deposition in a community repository (e.g. GitHub). See the Nature Portfolio guidelines for submitting code & software for further information.

Data

Policy information about availability of data

All manuscripts must include a data availability statement. This statement should provide the following information, where applicable:

- Accession codes, unique identifiers, or web links for publicly available datasets
- A description of any restrictions on data availability
- For clinical datasets or third party data, please ensure that the statement adheres to our policy

All biochemical data (Figs. 1b, c, d, 2, 3a, b, c, e, f, g, and 4d, Extended Figs. 1-4, 7b, 8-16, 17b and 18-20) generated in this study are provided in the source data file. The other data supporting the findings of this study are available from the corresponding author on reasonable request.

Field-specific reporting

Please select the one below that is the best fit for your research. If you are not sure, read the appropriate sections before making your selection.

☒ Life sciences ☐ Behavioural & social sciences ☐ Ecological, evolutionary & environmental sciences

For a reference copy of the document with all sections, see nature.com/documents/nr-reporting-summary-flat.pdf

Life sciences study design

All studies must disclose on these points even when the disclosure is negative.

Sample size	No statistical methods were used to predetermine sample size. The experiments in Fig 2d, Extended Fig 7d and 13 were performed in duplicate and the other all experiments were performed in triplicate. We believe all sample size were sufficient for the further analysis.
Data exclusions	No data were excluded from the analyses.
Replication	The experiments in figures 4, extended figures 1, 2, 5, 6, 7, 15, 17-20 were repeated at least twice and we confirmed the reproducibility of data as described in the manuscript. The other experiments were performed with triplicate or duplicate as shown in Sample size section.
Randomization	The experiments were not randomized. This study does not involve animals or human research participants.
Blinding	Investigators were not blinded to allocation during experiments and outcome assessment. This study does not involve animals or human research participants.

Reporting for specific materials, systems and methods

We require information from authors about some types of materials, experimental systems and methods used in many studies. Here, indicate whether each material, system or method listed is relevant to your study. If you are not sure if a list item applies to your research, read the appropriate section before selecting a response.

Materials & experimental systems

n/a	Involved in the study
☒	☐ Antibodies
☐	☒ Eukaryotic cell lines
☒	☐ Palaeontology and archaeology
☒	☐ Animals and other organisms
☒	☐ Human research participants
☒	☐ Clinical data
☒	☐ Dual use research of concern

Methods

n/a	Involved in the study
☒	☐ ChIP-seq
☒	☐ Flow cytometry
☒	☐ MRI-based neuroimaging

Eukaryotic cell lines

Policy information about cell lines

Cell line source(s)	Sf9 cells (Thermo Fisher Scientific), HEK293T (American Type Culture Collection)
Authentication	No cell lines were authenticated.
Mycoplasma contamination	No contamination.
Commonly misidentified lines (See ICLAC register)	No cell lines are on the database of commonly misidentified lines
